# High salt intake activates the hypothalamic–pituitary–adrenal axis, amplifies the stress response, and alters tissue glucocorticoid exposure in mice

**DOI:** 10.1093/cvr/cvac160

**Published:** 2022-11-11

**Authors:** Hannah M Costello, Georgios Krilis, Celine Grenier, David Severs, Alicja Czopek, Jessica R Ivy, Mark Nixon, Megan C Holmes, Dawn E W Livingstone, Ewout J Hoorn, Neeraj Dhaun, Matthew A Bailey

**Affiliations:** Edinburgh Kidney, British Heart Foundation Centre for Cardiovascular Science, The Universtiy of Edinburgh, Edinburgh, EH16 4TJ, United Kingdom; Edinburgh Kidney, British Heart Foundation Centre for Cardiovascular Science, The Universtiy of Edinburgh, Edinburgh, EH16 4TJ, United Kingdom; Edinburgh Kidney, British Heart Foundation Centre for Cardiovascular Science, The Universtiy of Edinburgh, Edinburgh, EH16 4TJ, United Kingdom; Department of Internal Medicine, Division of Nephrology and Transplantation, Erasmus Medical Center, University Medical Center Rotterdam, 3000 CA Rotterdam, The Netherlands; Edinburgh Kidney, British Heart Foundation Centre for Cardiovascular Science, The Universtiy of Edinburgh, Edinburgh, EH16 4TJ, United Kingdom; Edinburgh Kidney, British Heart Foundation Centre for Cardiovascular Science, The Universtiy of Edinburgh, Edinburgh, EH16 4TJ, United Kingdom; Edinburgh Kidney, British Heart Foundation Centre for Cardiovascular Science, The Universtiy of Edinburgh, Edinburgh, EH16 4TJ, United Kingdom; Edinburgh Kidney, British Heart Foundation Centre for Cardiovascular Science, The Universtiy of Edinburgh, Edinburgh, EH16 4TJ, United Kingdom; Edinburgh Kidney, British Heart Foundation Centre for Cardiovascular Science, The Universtiy of Edinburgh, Edinburgh, EH16 4TJ, United Kingdom; Department of Internal Medicine, Division of Nephrology and Transplantation, Erasmus Medical Center, University Medical Center Rotterdam, 3000 CA Rotterdam, The Netherlands; Edinburgh Kidney, British Heart Foundation Centre for Cardiovascular Science, The Universtiy of Edinburgh, Edinburgh, EH16 4TJ, United Kingdom; Edinburgh Kidney, British Heart Foundation Centre for Cardiovascular Science, The Universtiy of Edinburgh, Edinburgh, EH16 4TJ, United Kingdom

**Keywords:** Stress, Cortisol, Glucocorticoid excess, Glucocorticoid receptor, Salt

## Abstract

**Aims:**

High salt intake is common and contributes to poor cardiovascular health. Urinary sodium excretion correlates directly with glucocorticoid excretion in humans and experimental animals. We hypothesized that high salt intake activates the hypothalamic–pituitary–adrenal axis activation and leads to sustained glucocorticoid excess.

**Methods and results:**

In male C57BL/6 mice, high salt intake for 2–8 weeks caused an increase in diurnal peak levels of plasma corticosterone. After 2 weeks, high salt increased *Crh* and *Pomc* mRNA abundance in the hypothalamus and anterior pituitary, consistent with basal hypothalamic–pituitary–adrenal axis activation. Additionally, high salt intake amplified glucocorticoid response to restraint stress, indicative of enhanced axis sensitivity. The binding capacity of Corticosteroid-Binding Globulin was reduced and its encoding mRNA downregulated in the liver. In the hippocampus and anterior pituitary, *Fkbp5* mRNA levels were increased, indicating increased glucocorticoid exposure. The mRNA expression of the glucocorticoid-regenerating enzyme, 11β-hydroxysteroid dehydrogenase Type 1, was increased in these brain areas and in the liver. Sustained high salt intake activated a water conservation response by the kidney, increasing plasma levels of the vasopressin surrogate, copeptin. Increased mRNA abundance of *Tonebp* and *Avpr1b* in the anterior pituitary suggested that vasopressin signalling contributes to hypothalamic–pituitary–adrenal axis activation by high salt diet.

**Conclusion:**

Chronic high salt intake amplifies basal and stress-induced glucocorticoid levels and resets glucocorticoid biology centrally, peripherally and within cells.

## Graphical Abstract

**Graphical Abstract:**
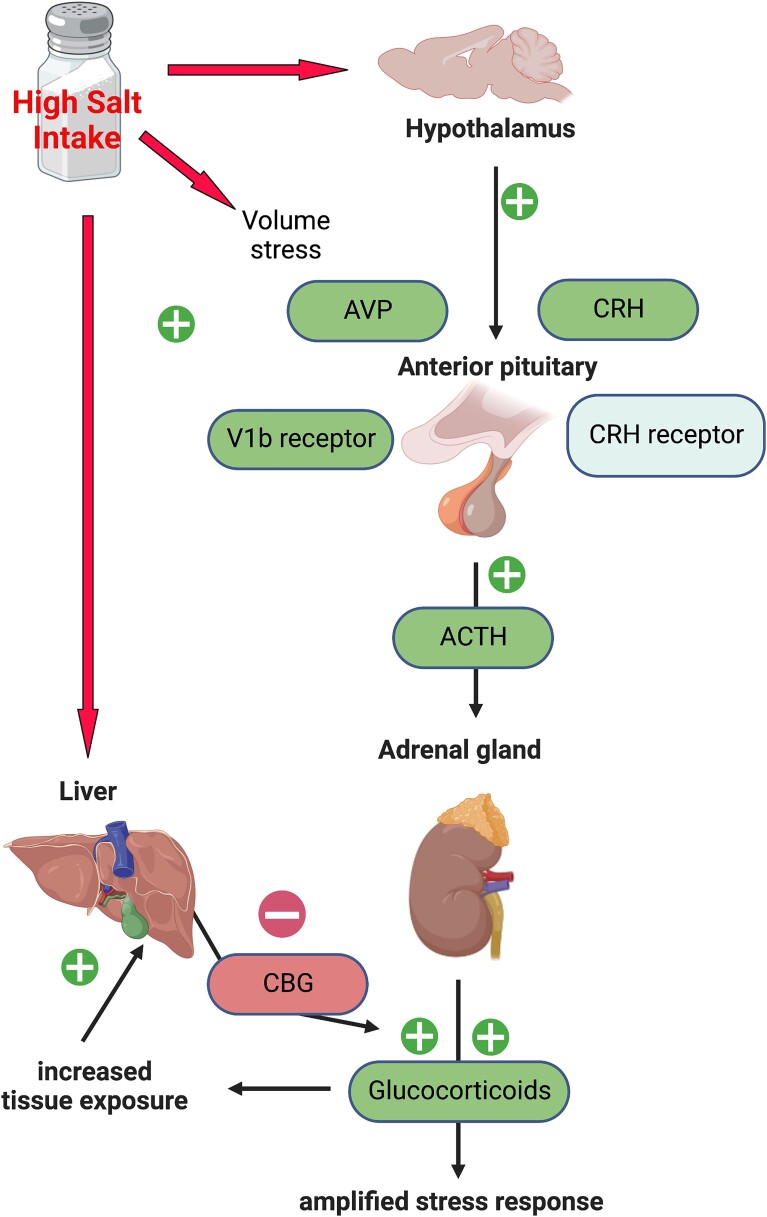

**See the editorial comment for this article ‘Salty secrets of the brain: the link between stress, salt, and hypertension’, by A.H. Ludwig-Słomczyńska and T.J. Guzik, https://doi.org/10.1093/cvr/cvad071.**

## 1. Introduction

The American Heart Association advocates a daily limit of 1500 mg Na (3.75 g salt) for most individuals, particularly those with hypertension.^1^ Daily salt intake in the United States of America, and most other countries, usually exceeds this threshold.^2^ In humans and experimental animals high salt intake induces abnormal physiological function of many organ systems and causes a range of poor health outcomes.^3^ This damaging effect of salt is most clearly seen in ‘salt-sensitive’ experimental models, i.e. those which display an exaggerated hypertensive response to high salt intake. Salt-sensitivity is also found in ∼30% of healthy humans and independently increases cardiovascular risk^4^ and mortality risk.^5^ Nevertheless, even in those people categorised as salt-resistant, high salt intake modifies organ function and accrual of incremental deficits over time will cause disease. Thus habitual dietary salt excess causes cardiovascular disease and may contribute to the development of autoimmunity,^6^ some cancers^7^ and cognitive impairment.^8^ High dietary salt clearly exerts a significant global health burden. Interventions to reduce intake improve outcomes.^9^ However, such reductions are difficult to achieve and sustain in the general population. Understanding how cell and organ physiology responds to chronic high salt intake offers an additional route to improve health across an array of diseases.

Our study focuses on glucocorticoids (cortisol in humans, corticosterone in rodents), powerful hormones which underpin many important cardiovascular, cognitive, immune, and metabolic cell functions. Glucocorticoids are not normally considered key regulators of salt balance. Nevertheless, observational studies in humans show a positive correlation between urinary free cortisol excretion and 24 h sodium excretion, taken to reflect salt intake.^3,10–12^ A small number of controlled sodium intake studies, typically lasting ∼7 days, have examined the relationship between salt intake and urinary excretion of glucocorticoids. Ranging from 8 to >600 subjects, these studies consistently report a direct relationship between dietary salt intake and urine cortisol excretion.^13–19^ A long-term balance study also found a positive relationship between salt intake and urinary cortisol excretion in healthy men enrolled in the MARS-500 spaceflight simulation programme.^20^ The direct association between salt intake and urinary glucocorticoid excretion has also been reported in mice^19,21,22^ and is exaggerated in mice with reduced expression of the glucocorticoid metabolizing enzyme 11β-hydroxysteroid dehydrogenase Type 2 (11βHSD2), which have salt-sensitive blood pressure.^21^

Based on these associations, we hypothesized that sustained high salt intake would activate the hypothalamic–pituitary–adrenal (HPA) axis. In male C57BL/6 mice, we find that salt intake increases basal and stress-induced corticosterone levels and alters aspects of peripheral glucocorticoid biology.

## 2. Methods

Experiments were performed between September 2018 and June 2022. Adult male C57BL/6 mice were commercially sourced (Charles River, UK) at age 10–12 weeks and maintained under controlled conditions of temperature (24 ± 1⁰C), humidity (50 ± 10% humidity) and light (lights on 7am to 7pm local time). Mice had *ad libitum* access to water and commercial rodent chow. The control diet contained 0.3% sodium and 0.7% potassium by weight [RM1, SDS Diets, United Kingdom (UK)]; the high salt diet contained 3% sodium and 0.6% potassium by weight (RM 3% Na^+^ SY, SDS Diets, UK). Mice were randomized into treatment groups and experiments performed with a single blinding to group allocations. No anaesthetic agents were used in these experiments and animals were euthanised either by cervical dislocation or by decapitation. All procedures conform to the guidelines from Directive 2010/63/EU of the European Parliament on the protection of animals used for scientific purposes. All experiments were performed in accordance with the UK Animals (Scientific procedures) Act under a UK Home Office Project Licence to the lead investigator (MAB) following ethical review by the University.

See Supplementary material online for Expanded Methods.

### 2.1 Basal HPA axis activity

Corticosterone was measured at the diurnal nadir (7am) and peak (7pm) in mice fed either high salt or control diet for up to 8 weeks. At the end of these experiments, mice were killed between 7 and 8am, and tissue was taken for mRNA measurements.

### 2.2 HPA axis response to stress

Each animal mouse was removed from its home cage between 7 and 8am and blood collected by tail venesection to give baseline corticosterone. Each mouse was then restrained in a Plexiglas tube for 15 min and another blood sample taken to measure the peak corticosterone response. Each mouse was then returned to its home cage. The protocol permitted collection of one further blood sample from each mouse, taken at either 30−, 60− or 90-min post-restraint stress.

### 2.3 Corticosterone, copeptin and aldosterone measurement

Corticosterone was extracted from tail venesection samples and measured by commercial ELISA kit (ADI-900-097; Enzo Life Sciences, UK). For copeptin and aldosterone, mice were euthanized by decapitation (between 7am and 8am), trunk blood was collected on ice, and separated by centrifugation and stored at −20⁰C. After a single thaw of all samples, copeptin (CEA365Mu; Cloud-Clone Corp., USA) and aldosterone (ADI-900-173; Enzo Life Sciences, UK) were measured by commercial ELISA.

#### 2.3.1 Copeptin and aldosterone measurement

Mice were euthanized by decapitation (between 7am and 8am), trunk blood was collected on ice, and separated by centrifugation and stored at −20⁰C. After a single thaw of all samples, copeptin (CEA365Mu; Cloud-Clone Corp., USA) and aldosterone (ADI-900-173; Enzo Life Sciences, UK) were measured by commercial ELISA.

### 2.4 Corticosterone-binding globulin binding capacity

This was measured using a saturation binding assay, as described,^23^ with the maximal binding capacity (Bmax), estimated using non-linear regression.

### 2.5 Quantitative polymerase chain reaction

mRNA abundance in tissues was measured by quantitative RT-PCR using the Universal Probe Library (Roche, UK) or PowerUp SYBR Green (Applied Biosystems, UK). Primer sequences and probe numbers are given in Supplementary material online, *Table S1*. The results were normalized to the mean concentration of reference genes. These were *Actb* and *Tbp* for adrenal, hippocampus and anterior pituitary; *Actb* and *Hprt* for liver; *Actb* and *Rn18s* for kidney cortex/medulla and aorta; *Actb* and *Gapdh* for heart; *Gapdh* and *Tbp* for hypothalamus. *Before normalization, the concentration of mRNA for reference genes in hypothalamus, liver, kidney and heart from mice fed control diet (n = 8) and mice fed high salt diet for 2 weeks (n = 8) was compared by Mann–Whitney U-test. There was no effect of salt intake*: *P*-*values ranged from P = 0.181 to P = 0.993*

### 2.6 Quantification and statistical analysis

Group size estimates were based on pilot experiments demonstrating an increase in peak plasma corticosterone after 2 weeks of high salt intake. Power analysis was performed using G*Power v3.19 Software,^24^ using a main effect alpha level of *P* < 0.05 and 90% power. No animals were excluded from study, but some samples subsequently failed quality control in assay (e.g. for RNA quality/abundance; blood sample volume) and retrospective power analysis on data sets was therefore performed. In final data sets, individual points are shown along with group mean ± SD. Statistical analysis was performed using GraphPad Prism v8.4 Software. The distribution of data sets was assessed using the Shapiro–Wilk normality test. Normally distributed data sets were analysed by *t*-test, one-way ANOVA or two-way ANOVA, with or without repeated measures, as stated in the figure legends. For ANOVA, Holm–Sidak post-tests were used for planned comparisons. For data that did not pass normality testing, non-parametric analysis was carried out using Wilcoxon test. The sample number (*n*) and statistical analysis details used are stated in the figure legends; absolute *P*-values for planned comparisons are reported.

## 3. Results

### 3.1 High salt activates the HPA axis to increase corticosterone synthesis

**Figure 1:**
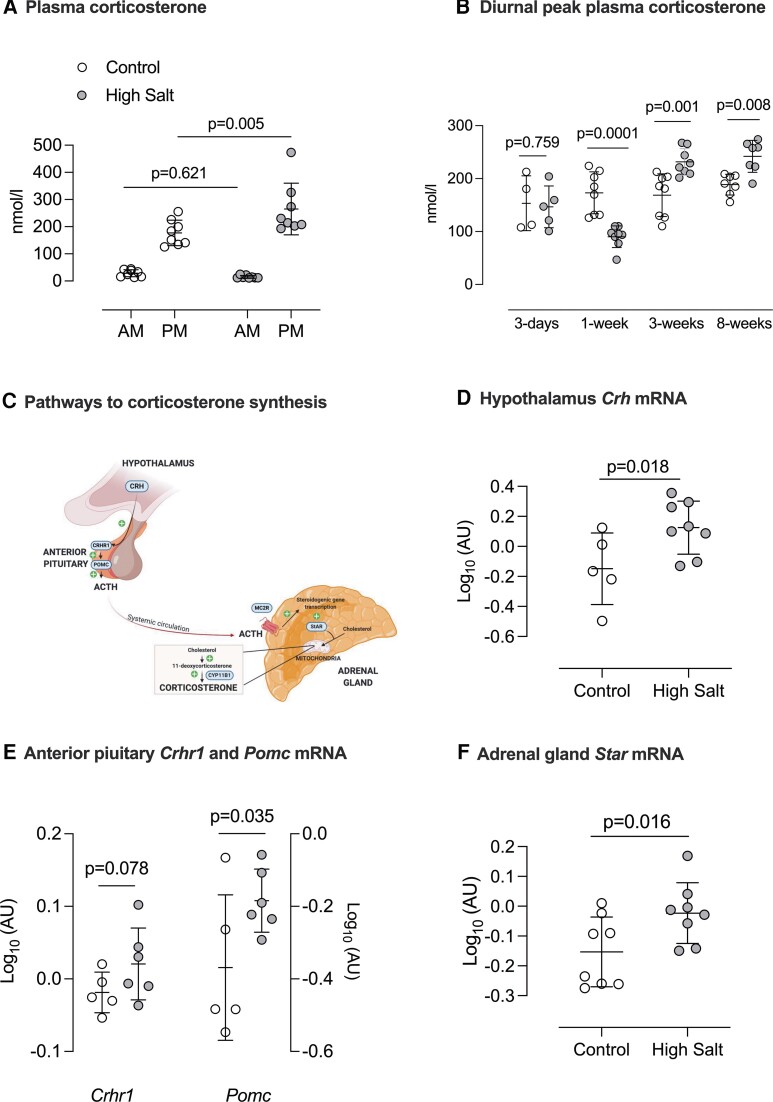
Activation of HPA axis after high salt intake. (*A*) Plasma corticosterone measured at 7am (diurnal nadir) and 7pm (diurnal peak) in male C57BL/6 mice (*n* = 8) first on a 0.3% Na diet (control, open circles) and again after 14 days on 3% Na diet (high salt; closed circles). Individual values are shown with group mean ± SD; statistical comparison was two-way repeated measures ANOVA for the main effects of time of day (*P* < 0.0001) and diet (*P* = 0.071) and the interaction (*P* = 0.016); *P*-values for planned comparisons (Holm–Sidak) are given. (*B*) The effect of salt diet on diurnal peak plasma corticosterone was measured in separate groups of mice fed either control (open circles) or high salt diet (closed circles) for 3 days (*n* = 4/5), 1 week (*n* = 8/8), 3 weeks (*n* = 8/8) or 8 weeks (*n* = 8/8) of either. Individual values are shown with group mean ± SD; statistical comparison was two-way ANOVA for the main effects of ‘*diet*’ (*P* = 0.476) and ‘*duration*’ (*P* < 0.0001) and the interaction (*P* < 0.0001); *P*-values for planned comparisons (Holm–Sidak) are shown. (*C*) Schematic highlighting key components of HPA axis activation that were then measured at mRNA level in tissue taken from different C57BL/6 mice fed either 0.3% Na (open circles *n* = 8) or 3%Na (closed circles *n* = 8) for 2 weeks. For hypothalamus and anterior pituitary, *n* = 5 for control diet and *n* = 6 for high salt diet following exclusion of samples because of poor mRNA yield/quality. (*D*) *Crh* (corticosterone releasing hormone) mRNA in hypothalamus (*E*) *Crhr1* (CRH receptor and *Pomc* (pro-opiomelanocortin) mRNA in anterior pituitary; and (*F*) adrenal gland *Star* (Steroidogenic Acute Regulatory protein) mRNA. Individual values are shown with group mean ± SD; statistical comparison was by unpaired *t*-test with *P*-values as indicated.

The hypothesis that high salt intake activated the HPA axis was initially tested in *n* = 8 mice, sampling blood at 7am and 7pm to capture the diurnal nadir and peak of corticosterone. Measurements were made in each mouse on control diet (0.3% Na) and again after 2 weeks of high salt (3% Na). High salt intake significantly increased peak plasma corticosterone, without affecting the nadir (*Figure 1A*). We next examined the time course, measuring peak corticosterone in separate groups of mice maintained on high salt or control diets for 3 days, 1−, 3−, and 8-weeks (*Figure 1B*). The effect of salt intake was biphasic: at 1-week, peak corticosterone was suppressed, after which high salt intake increased peak corticosterone levels, and this stimulatory effect was sustained.

Using tissue taken from the 2-week high salt group, we examined mRNA expression for key genes leading to increased corticosterone production (*Figure 1C*). Hypothalamic *Crh* expression (encoding corticotropin releasing hormone; CRH) was elevated (*Figure 1D*). The mRNA abundance of the anterior pituitary CRH receptor, *Crhr1*, tended to be higher in the high salt group and pro-opiomelanocortin, the precursor of adrenocorticotropic hormone (ACTH), was significantly increased by high salt intake (*Figure 1E*). Adrenal gland weight and the mRNA levels of 11β-hydroxylase and the ACTH receptor were not changed by high salt diet (see Supplementary material online, *Figure S1*) but mRNA encoding steroidogenic acute regulatory protein (StAR) was increased (*Figure 1F*). StAR expression, the regulated rate-limiting step for adrenal steroid biosynthesis, is increased chronically by ACTH.^25^ ACTH can also stimulate aldosterone excretion^26^ but here we found broad suppression of the renin–aldosterone system by high salt intake: mRNA for hepatic angiotensinogen (*Agt*), kidney renin (*Ren*) and adrenal gland aldosterone synthase (*Cyp11b2*) were all reduced and plasma aldosterone was supressed. Additionally, renal mRNA expression of the mineralocorticoid receptor was significantly downregulated in mice on a high salt diet (see Supplementary material online, *Figure S2*).

**Figure 2:**
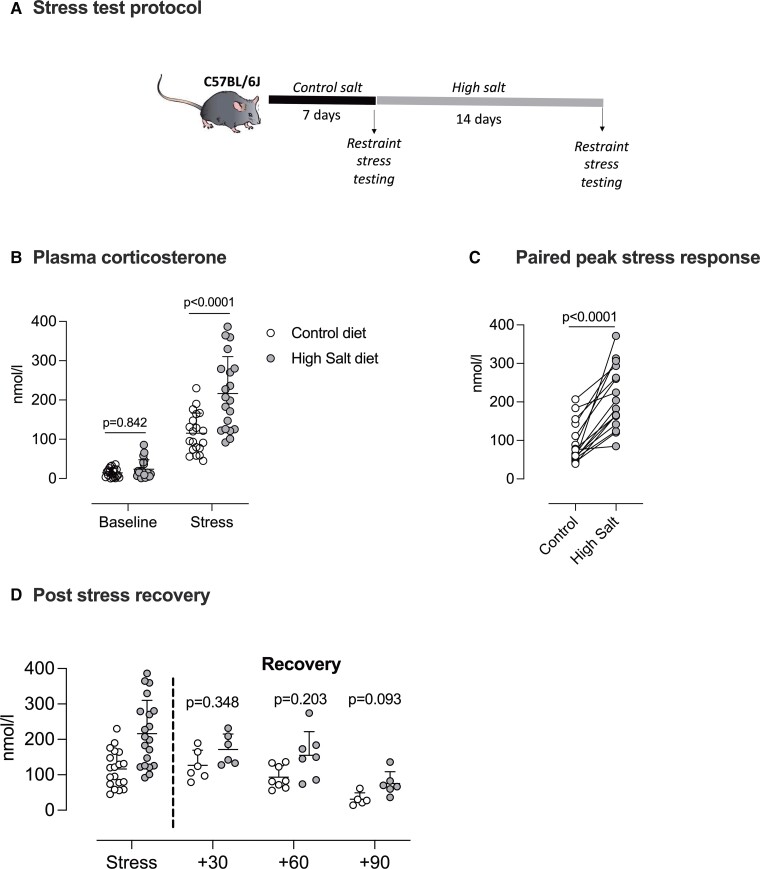
High salt intake amplifies the stress response. (*A*) protocol for measuring the stress response in male C57BL/6 mice (*n* = 20) fed 0.3% Na diet (control; open circles) and again after 2 weeks on a 3% Na diet (high salt; closed circles). (*B*) Plasma corticosterone in samples taken at 8am (baseline) and again after 15 min of tube restraint (Stress). Analysis was two-way ANOVA with repeated measures for the main effects of ‘*stress-response*’ (*P* < 0.0001) and ‘*diet*’ (*P* = 0.0006) and the interaction (*P* = 0.0014); *P*-values for planned comparisons (Holm–Sidak) are given. (*C*) The paired peak stress response in each mouse (*n* = 20), analysed by paired *t*-test. (*D*) The corticosterone recovery from stress was measured at either 30 (*n* = 6/6), 60 (*n* = 8/7) or 90 minutes (*n* = 5/6) following release from the restraint tube. Analysis was two-way ANOVA with repeated measures for the main effects of ‘*time*’ and ‘*diet*’ and the interaction; *P*-values for planned comparisons (Holm–Sidak) are given. Individual measurements are shown with group mean ± SD.

In a different group of mice (*n* = 20), we assessed the effect of high salt intake on the HPA axis response to a 15 min restraint stress (*Figure 2A*). On control diet, restraint stress increased plasma corticosterone in all mice, with a group average increase of ∼90 nmol/L (*Figure 2B*). After 2 weeks of high salt intake, the peak stress response was amplified in 16 of these mice (*Figure 2C*), and the group mean response approximately doubled.

Protocol welfare considerations permitted only one additional blood sample per mouse, and this was drawn by tail venesection at either 30−, 60−, or 90-minutes post-restraint (*Figure 2D*). Assessed by ANOVA at group level, the main effects of time (*P* < 0.0001) and salt diet (*P* = 0.007) were significant, but the interaction was not (*P* = 0.344), and there were no significant differences in the planned *post-hoc* comparisons, indicating that the rate of recovery from stress was not affected by salt intake.

### 3.2 High salt reduces corticosterone-binding globulin expression and capacity

**Figure 3:**
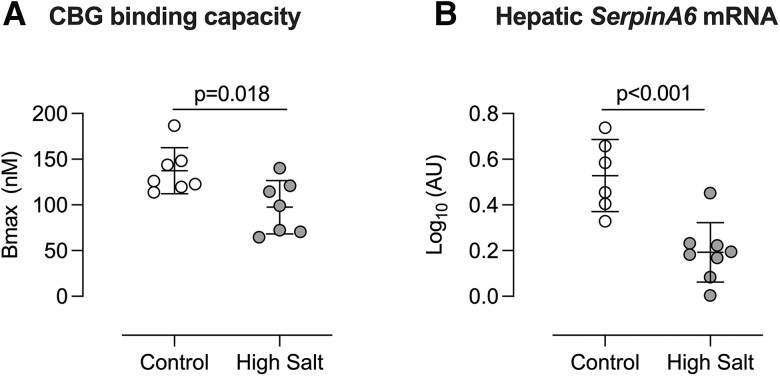
Effect of high salt intake on corticosterone-binding globulin. Male C57BL/6 mice were fed either 0.3% (control; open circles) or 3% (high salt; closed circles), diet for 14 days and blood taken for measurement of (*A*) Corticosterone-binding globulin binding capacity (*n* = 7/7). (*B*) mRNA abundance of *SerpinA6*, encoding CBG, was measured in liver cDNA (*n* = 6/8). Expression was normalized to that of reference genes *Gapdh* and *Tbp* and log-transformed. Individual measurements are shown with group mean ± SD; statistical comparisons were made with Student’s unpaired *t*-test and two-tailed *P*-values are given.

Although high salt diet increased the total amount of circulating corticosterone at rest and under stress, most glucocorticoid is bound to corticosterone-binding globulin (CBG) and only unbound hormone is active. Thus, CBG has the potential to act as a buffer, stabilising the unbound, active fraction against increased hormone production. We therefore measured CBG binding capacity, which was significantly reduced after 14 days of high salt intake (*Figure 3A*). Liver mRNA levels of the encoding gene, *SerpinA6*, was also lower in the high salt group (*Figure 3B*). *SerpinA6* expression was also significantly reduced in mice after 7 days of high salt intake (see Supplementary material online, *Figure S3*), although CBG binding capacity was not different from the control group at this time point.

### 3.3 High salt alters glucocorticoid receptor signalling in tissues

**Figure 4:**
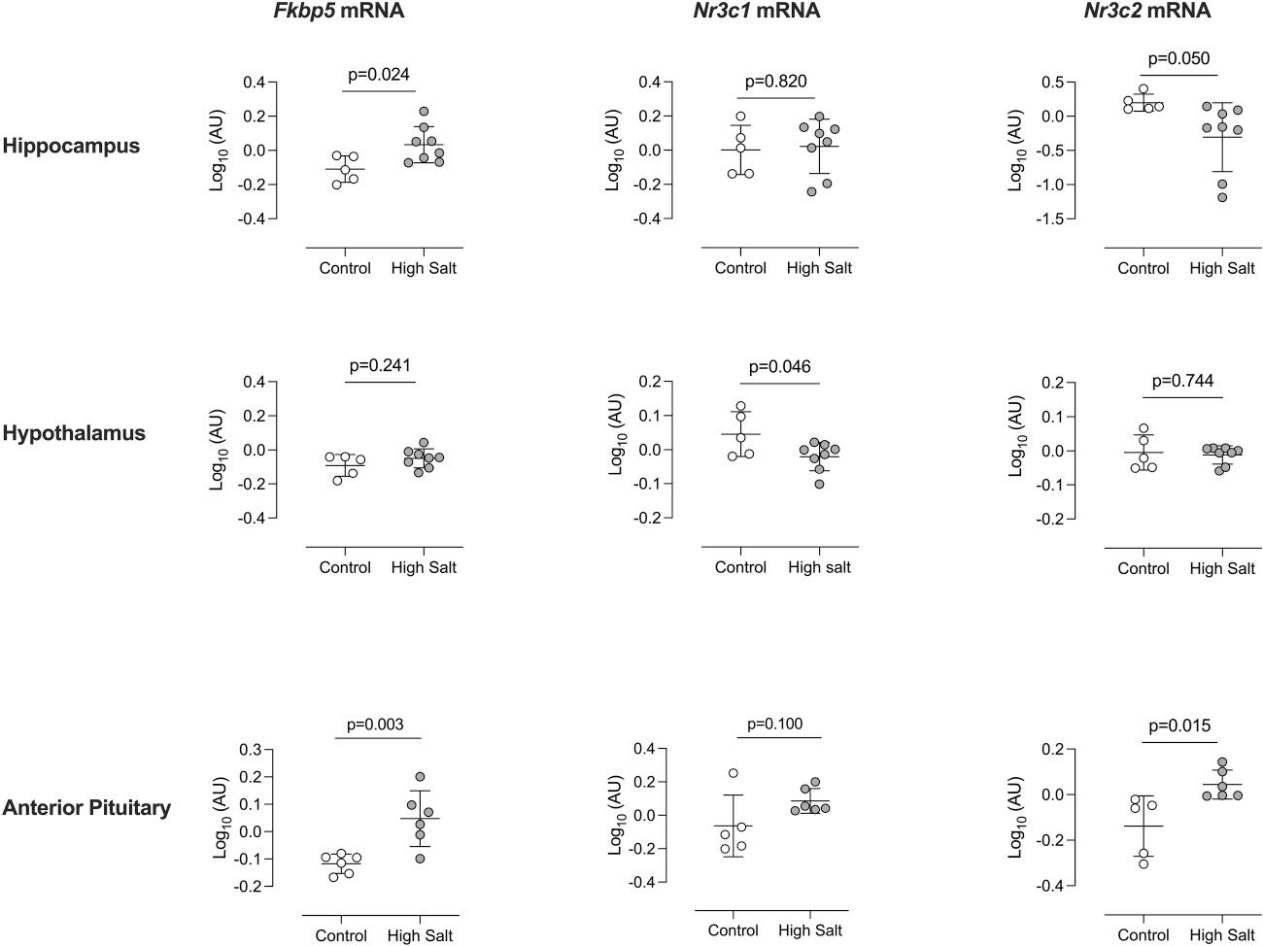
Effect of high salt intake on mRNA abundance of *Fkbp5*, *Nr3c1* and *Nr3c2* in brain regions. Male C57BL/6 mice were fed either 0.3% (control; open circles; *n* = 8) or 3% (high salt; closed circles; *n* = 8), diet for 14 days and tissues taken at cull. Excluding samples that failed to yield sufficient material for analysis, mRNA abundance of FK506-binding protein 5 (*Fkbp5*), glucocorticoid receptor (*Nr3c1*) and mineralocorticoid receptor (*Nr3c2*) was measured in hippocampus (*n* = 5/8), hypothalamus (*n* = 5/8) and anterior pituitary (*n* = 5/6) Values were normalized to the expression of reference genes *Gapdh* and *Tbp* and log-transformed. Individual measurements for samples that passed QC are shown, along with group mean ± SD. Statistical comparisons were made with Student’s unpaired *t*-test and *P*-values are given.

We hypothesised that these effects of high salt diet would combine to increase glucocorticoid signalling within tissues. Glucocorticoid exposure, reported by mRNA encoding FK506-binding protein 5 (*Fkbp5)*^27^ was increased in the hippocampus and anterior pituitary but not in the hypothalamus, where mRNA abundance for the glucocorticoid receptor (*Nr3c1*) was significantly reduced (*Figure 4*). In the anterior pituitary, mRNA abundance for the glucocorticoid receptor (*Nr3c1*) tended to be higher in mice fed high salt, but this was not statistically significant; mRNA for the mineralocorticoid receptor (*Nr3c2*) was increased with high salt. In the hippocampus, receptor expression was not strongly influenced by dietary salt but the enzyme 11β-hydroxysteroid dehydrogenase Type 1 (11βHSD1), which regenerates active glucocorticoid in cells to amplify glucocorticoid signalling,^28^ was upregulated at mRNA level (see Supplementary material online, *Figure S4*).

In peripheral tissues, *Fkbp5* mRNA was upregulated in the liver, despite downregulation of the glucocorticoid receptor; 11βHSD1 expression was again upregulated (see Supplementary material online, *Figure S5*). In contrast, kidney *Fkbp5* mRNA was downregulated in cortex and medulla, as was mineralocorticoid receptor (*Nr3c2*) mRNA expression; the glucocorticoid receptor (*Nr3c1*) was downregulated in only the kidney cortex (see Supplementary material online, *Figure S2* and *S6*). There were no significant changes in these transcripts in the heart, aorta (see Supplementary material online, *Figure S7*) or adrenal gland (see Supplementary material online, *Figure S8*) after 2 weeks of high salt intake.

### 3.4 High salt intake and water conservation

**Figure 5:**
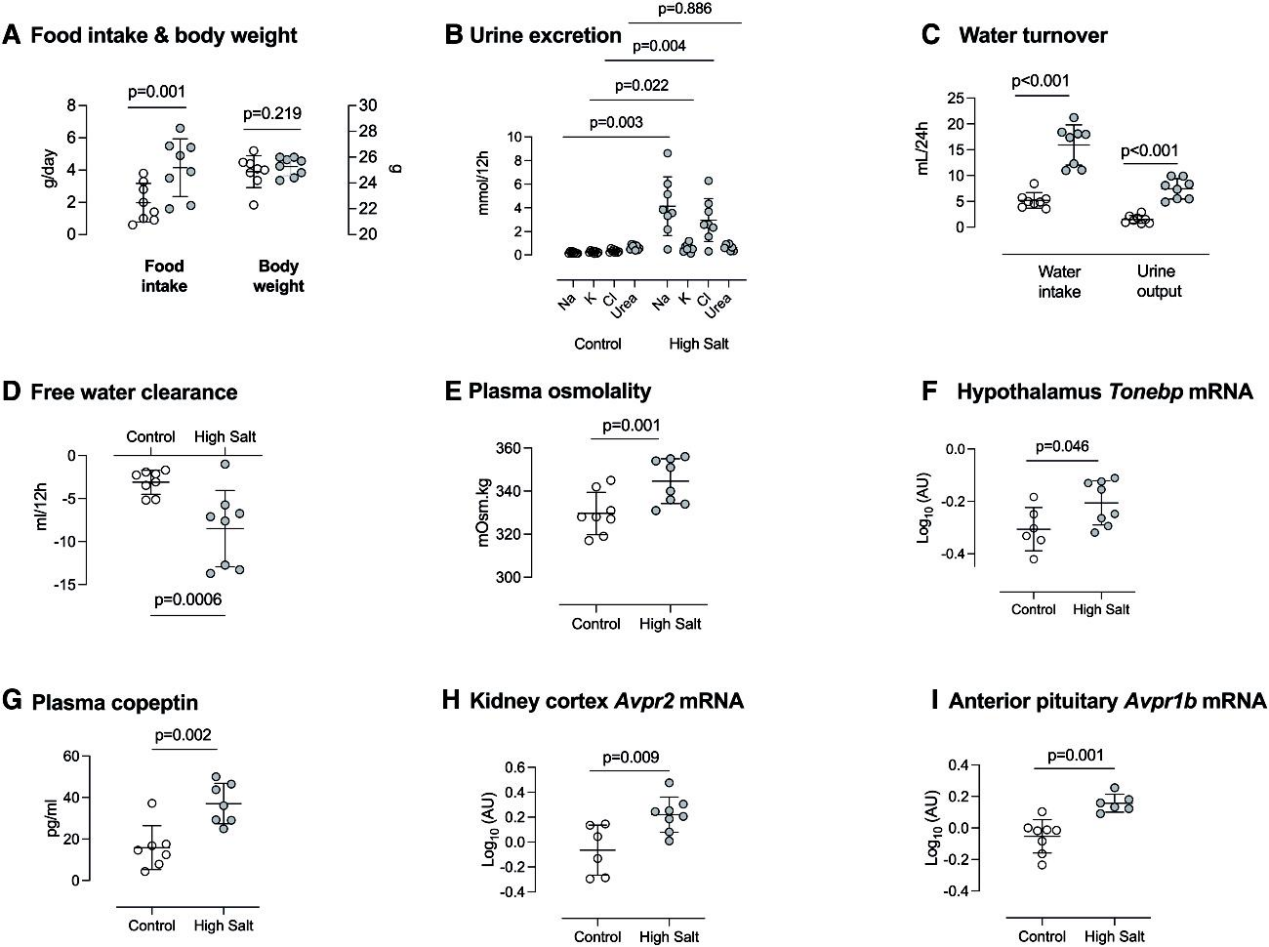
Effect of high salt intake on fluid volume markers. Male C57BL/6 mice were fed 0.3% (Control; open circles *n* = 8) diet and then 3% (high salt; closed circles *n* = 8) diet for 14 days. (*A*) Food intake and body weight; (*B*) urine excretion of sodium, potassium, chloride and urea; (*C*) water turnover; (*D*) free water clearance and (*E*) plasma osmolarity. The following measurements were made from samples collected from other experiments: (*F*) mRNA abundance of tonicity-responsive enhancer-binding protein (*Tonebp*) in the hypothalamus; (*G*) plasma copeptin; (*H*) mRNA abundance of vasopressin V2 receptor (*Avpr2*) in kidney cortex and (*I*) mRNA abundance of vasopressin V1b receptor (*Avpr1b*) in anterior pituitary. Individual measurements are shown with group mean ± SD; statistical comparisons were made with Student’s unpaired *t*-test and two-tailed *P*-values are given.

Recent studies indicate that a high salt diet causes a metabolic shift to produce organic osmolytes, which help stabilize body water in the face of salt-induced diuresis.^22,29^ We examined this in mice fed high salt or control diet for 2 weeks. High salt diet increased food intake but body weight did not change over the 2-week experimental timeframe (*Figure 5A*). As expected, both urinary sodium, chloride and potassium excretion were significantly increased, but urinary urea excretion was not affected by high salt intake (*Figure 5B*). Plasma urea concentration was modestly increased by high salt intake but the mRNA abundance for rate-limiting enzymes for ureagenesis and gluconeogenic enzymes (pyruvate carboxylase and phosphoenolpyruvate carboxykinase 1) were not significantly changed in either kidney or liver (see Supplementary material online, *Figure S9*).

High salt diet induced polyuria and polydipsia (*Figure 5C*). Free water clearance was significantly reduced (*Figure 5D*) and sustained high salt intake increased plasma osmolality (*Figure 5E*) and plasma sodium concentration (see Supplementary material online, *Figure S9*). Other evidence was indicative of a water conservation response. In the hypothalamus, mRNA encoding Tonicity-responsive enhancer-binding protein (TonEBP), which reflects parallel transcription/translation of vasopressin in response to hypertonicity^30^ was increased by high salt (*Figure 5F*). Plasma copeptin, a stable surrogate for vasopressin secretion,^31^ was also elevated (*Figure 5G*), as was mRNA abundance of the vasopressin V2 receptor in the kidney (*Figure 5H*). Vasopressin can also activate V1b receptors in the anterior pituitary to stimulate ACTH release,^32^ and this receptor was upregulated at the mRNA level in mice fed high salt diet (*Figure 5I*).

## 4. Discussion

The main finding of our study is that sustained high dietary salt intake in mice induced multiple abnormalities in glucocorticoid biology. The HPA axis was activated and the response to environmental stress amplified. The capacity of CBG to buffer a rise in blood corticosterone was diminished, and in the hippocampus, anterior pituitary and liver, exposure to glucocorticoids was increased.

Our study provides a mechanistic explanation of the positive correlation between salt intake and urinary cortisol excretion consistently observed in humas,^12–18,20^ and has two important implications. First, glucocorticoid excess may contribute to the long-term health consequences of high salt intake; second, dietary salt intake becomes an important consideration when diagnosing and managing pre-existing hypercortisolism.

### 4.1 HPA axis activation at rest and in response to stress

We show functional evidence of a novel, direct connection between dietary salt intake and HPA axis activation. The effect was biphasic: diurnal peak plasma corticosterone was supressed at 1 week and thereafter significantly enhanced. In humans, 1 week of high salt also reduces plasma cortisol, which may reflect an initial phase of enhanced urinary elimination.^33^ Longer-term, however, our data indicate that high salt intake increases corticosterone production: hypothalamic CRH mRNA was increased and peak plasma levels were elevated. A sustained increase in circulating glucocorticoid, should normally downregulate CRH receptor mRNA expression in the anterior pituitary.^34,35^ Such downregulation did not happen in our study and *Crhr1* mRNA abundance was, overall, higher in the high salt group compared with controls. Combined with significantly increased *Pomc* mRNA in anterior pituitary, our data indicates enhanced ACTH production/release with sustained high salt intake. Our results suggest two plausible explanations for this phenomenon. First, it is possible that high salt intake disrupts the normal negative feedback control of the HPA axis by corticosterone. In the hippocampus, this negative feedback occurs via the mineralocorticoid receptor because 11HSD2 is not expressed.^36^ High salt reduced mineralocorticoid receptor mRNA levels, which, on the basis of previous genetic and pharmacological studies, would be expected to enhance basal HPA axis activity.^37^ That high salt increased anterior pituitary *Crh1* mRNA is also consistent with impaired negative feedback.^34,35^

A second possibility is that vasopressin, a co-regulator of ACTH secretion,^32^ contributes to enhanced glucocorticoid release. In support of this hypothesis, high salt intake increased serum copeptin, which quantitatively reports hypothalamic synthesis/release of vasopressin.^31^ High salt also upregulated mRNA for the vasopressin V1b receptor in the anterior pituitary and although this might not fully parallel receptor number,^38^*V1br* knockout mice have reduced basal corticosterone and an attenuated response to acute stress.^39^ Glucocorticoids also directly increase mRNA *V1br* mRNA abundance and enhance receptor coupling to phospholipase C.^40^ Overall, a role for vasopressin is a plausible interpretation of our data and is discussed more below.

Amplification of the stress response was found in this study. We do not think this reflects the repeated sampling as the protocol does not itself increase basal corticosterone, cause stress-induced changes in the leukogram or cause stress-associated changes in mouse behaviour.^41^ We conclude that it is the salt intake itself that enhances the stress response and in this context, we have previously found that high salt diet activates the sympathetic nervous system activation in C57BL/6 mice.^42^ Indeed, high salt diet is emerging as an important behavioural modifier, at least in rodents.^43^ This warrants systematic investigation because detrimental changes in brain health in response to high dietary salt intake may have important real-world consequences.

### 4.2 Peripheral glucocorticoid homeostasis

After two weeks of high salt feeding, total CBG binding capacity for corticosterone was reduced. This reflects early downregulation of *Serpina6* mRNA, which after several days reduces the amount of CBG in circulation.^44^ Whether high salt diet modulates molecular binding affinity of CBG for corticosterone, through altered glycosylation for example^45^ remains undetermined. The consequences of reduced CBG binding are difficult to gauge. On one hand, CBG is a reservoir for corticosterone in circulation and enhanced glucocorticoid signalling might therefore be anticipated. On the other hand, CBG also contributes to tissue delivery of glucocorticoid^46^ and CBG-deficient mice are hyporesponsive to corticosterone.^47^

Our data do not indicate a consistently altered glucocorticoid response after two weeks of high salt intake. Elevated *Fkbp5* mRNA suggests glucocorticoid exposure was enhanced in liver, anterior pituitary and hippocampus, reduced in the kidney and unchanged in other tissues examined. The overall picture is complex because changes to the glucocorticoid and mineralocorticoid mRNA expression were accompanied by evidence of altered intracellular glucocorticoid regeneration *via* 11βHSD1. This granularity is important and shows that high salt intake is influencing glucocorticoid biology at both the systemic and cellular level. Importantly, local changes in glucocorticoid regeneration alter high-level physiological phenotypes.^48^ For example, transgenic 11βHSD1 overexpression in liver^49^ and adipose^50^ cause metabolic abnormalities with salt-sensitive hypertension; overexpression in the hippocampus accelerates age-related cognitive decline.^51^

### 4.3 High salt, glucocorticoids, and water homeostasis

Recent studies show that high salt intake engages a water conservation response akin to aestivation, the metabolic torpor induced in diverse species through extended exposure to a hot and arid environment.^52^ Central to this response is a shift to muscle catabolism, accumulating organic osmolytes such as urea within the body to stabilize fluid volume during extreme challenges to water balance. Thus, in mice facing the dehydrating challenge of high salt diet combined with 0.9% saline as the sole source of fluid, gluconeogenesis and ureagenesis is evident at 2 weeks, associated with an increase in plasma corticosterone.^22^ This association is also found in humans,^19,29^ suggesting that glucocorticoids may drive the metabolic reprioritisation, although other clinical studies suggest that the energy demand imposed by glomerular hyperfiltration is more important.^12^

Our study was not designed to interrogate the metabolic response to high salt intake, but we can make some limited inferences. For example, body weight did not increase despite higher calorific intake and plasma urea was higher in mice fed high salt, suggesting catabolism. On the other hand mRNA abundance of pyruvate carboxylase and phosphoenolpyruvate carboxykinase 1, used to report gluconeogenic flux in liver and kidney^53^ were not changed by high salt diet, although mRNA levels are not the only factor.^54^

Certainly, despite free access to water, 2-weeks of high salt diet induced plasma hypertonicity and hypernatremia, indicating compromised fluid balance. Indeed, our observations indicate activation of a water conservation response: hypothalamic mRNA abundance of TonEBP (which parallels vasopressin production in response to hypertonicity^30^) was increased by high salt intake, as was circulating copeptin (reporting vasopressin synthesis^31^). Finally, in the kidney, V2 receptor mRNA levels, which parallels protein expression and receptor activity^55^ were significantly increased, supporting enhanced water reabsorption in the collecting duct when high salt intake is high.^56^

It is possible that the need to activate an extreme water conservation response underpins the rise in glucocorticoids that will ultimately mobilise organic osmolytes. Increased vasopressin synthesis, upregulation of the V1b receptor in the anterior pituitary^39^ and CRH in the hypothalamus^57^ will synergise to activate the HPA axis and elevate circulating glucocorticoid.

### 4.4 Limitations

Our study aimed to investigate the hypothesis that high salt intake activated the HPA axis. In this, the study was successful and in describing the phenomenon, the data provide a rationale for detailed examination of the consequences for organ function. However, our study is limited. Most experiments provide a snap-shot view at two weeks. Aspects of our study are based on measurements of bulk tissue mRNA abundance for a small number of genes. Given the breadth of the glucocorticoid transcriptome and the challenges of connecting transcriptional changes to altered protein function, our study provides a restricted insight into the potential consequences of salt-induced HPA axis activation. Finally, although the association between urinary salt and glucocorticoid excretion is seen in both women and men, there is well-documented sexual dimorphism in HPA axis function at rest and during the response to stress.^58^ Our research capability was much reduced during the pandemic and we are not yet able to report a detailed examination of the relationship between salt intake and HPA axis function in female mice.

### 4.5 Perspectives

We find evidence of accumulated glucocorticoid excess with sustained high salt intake in male C57BL/6 mice. Given that human salt intake is habitually high, our results are relevant to human health. HPA axis induction by high salt intake may serve a critical role in preserving fluid balance but the long-term consequences of this are likely detrimental: glucocorticoid excess typically promotes sodium retention and salt-sensitive blood pressure abnormalities.^59–61^ HPA activation may also contribute to poor metabolic^10,62^ and cognitive health when salt intake is high.^63^

## Supplementary material

Supplementary material is available at *Cardiovascular Research* online.

## Author contributions

H.M.C., M.C.H., D.E.W.L., N.D., and M.A.B. contributed to the study design. H.M.C., G.K., C.G., A.C., and M.A.B. performed experiments. J.R.I. and M.N. designed protocols and provided reagents. All authors contributed to data production, statistical analysis, and interpretation. H.M.C., N.D., and M.A.B. wrote the initial draft of the manuscript; all authors reviewed and revised the manuscript.

## Supplementary Material

cvac160_Supplementary_Data

## Data Availability

The data underlying this article will be shared on reasonable request to the corresponding author

## Notes

Translational perspective The direct connectivity between salt intake and HPA axis function may be relevant for the long-term health consequences of a high salt diet.
