# Supplementary material for: High salt intake activates the hypothalamic–pituitary–adrenal axis, amplifies the stress response, and alters tissue glucocorticoid exposure in mice
Source: Cardiovasc Res. 2022 Nov 11;119(8):1740–50. doi: 10.1093/cvr/cvac160 (PMC10325699; doi:10.1093/cvr/cvac160)

Data Supplement for: CVR-2022-0300

High salt intake activates the hypothalamic-pituitary adrenal axis, amplifies the stress response and alters tissue glucocorticoid exposure in mice

Hannah M Costello[1], Georgios Krilis[1], Celine Grenier[1], David Severs[2], Alicja Czopek[1], Jessica R. Ivy[1], Mark Nixon[1], Megan C Holmes[1], Dawn E.W. Livingstone[1] Ewout J Hoorn[2,] Neeraj Dhaun[1,] Matthew A Bailey[1]*

[1]British Heart Foundation Centre for Cardiovascular Science, The University of Edinburgh, United Kingdom, EH16 4TJ, [2]

For Correspondence: Matthew Bailey, PhD, FRSB

Matthew.bailey@ed.ac.uk

Contents:

Expanded Methods

Table 1: primers and probes

Supplemental data figures 1-9

Expanded Methods

Blood sampling protocols. Mice were singly housed and blood collection by tail nick technique was performed just after the lights came on at 7am, coincident with the diurnal nadir of plasma corticosterone and just before the lights went off at ~7PM, coincident with the diurnal peak. Rapid collection is required. Mice were removed from their home cages and placed on top of an empty clean cage. The tip of the tail (<1mm) was snipped off using sharp sterile scissors. Mice were minimally restrained during blood sample collection and allowed to walk around the top of the cage until ~30μL of blood was collected into an EDTA coated tube and placed on wet ice. Blood was centrifuged at 2000 x g for 5 min at 4°C and plasma transferred to a fresh Eppendorf to be stored at -80°C for subsequent plasma corticosterone assays. A large plasma sample was needed to quantify aldosterone and copeptin concentrations and measure CBG binding capacity. Blood was taken post-mortem via decapitation, centrifuged at 2000 x g for 5 min and stored at -80°C until all samples were collected for subsequent assays*.*

Restraint stress testing. n=20 mice were used and singly housed. Each animal was removed from their home cage at ~7am and ~30μL of blood was collected within 1 minute of handling by tail venesection to give baseline corticosterone. Each mouse was then held in a Plexiglas restraint tube for 15 min, after which a second blood sample was drawn to provide the peak corticosterone response. The mouse was then returned to its home cage. We were permitted to take one further blood sample from each mouse, and we measured plasma corticosterone at either 30-, 60-, or 90-min post-restraint stress. In some animals, we were unable to obtain sufficient blood for analysis, giving n=5-7 at each of the time points. Statistical analysis of the post-restraint recovery phase was therefore performed independently of the initial stress response.

Metabolic cage protocol. Mice (n=8) were fed control diet for 6 days and then singly housed in metabolic cages for 24 h. Body weight was recorded, and food and water intake assessed over 24hrs. Urine was collected between 7pm and 7am. A ~30μL blood sample was drawn by tail venesection. Mice were returned to their home cage and fed high salt diet for 13 days. After this, body weight was recorded, and mice then returned to the metabolic cage for 24h as before. Plasma osmolality was measured in aliquots of plasma stored at -80C using by freezing-point depression (Type 15 Löser Osmometer, Camlab, Cambridge, UK). Plasma sodium concentration was measured by ion-selective electrode (model 9180, Roche Diagnostics, UK). Plasma urea was measured by colourmetry (Kit MAK006, Sigma Aldrich, UK). Plasma samples were diluted 1:50 and Urine samples 1:5000 (control) & 1:2000 (High salt diet).

Corticosterone measurement. Because of small sample volumes, plasma corticosteroneconcentration was quantified in duplicate using a corticosterone ELISA kit (ADI-900-097; Enzo Life Sciences, UK), with a lower limit of detection of 27pg/ml. (Enzo Life Sciences, Exeter, UK). Cross reactivity for this kit is as follows: corticosterone (100%), deoxycorticosterone (21.3%), desoxycorticosterone (21.0%), progesterone (0.46%), testosterone (0.31%), tetrahydrocorticosterone (0.28%), aldosterone (0.18%), cortisol (0.046%) and <0.03%: pregnenolone, estradiol, cortisone, 11-dehydrocorticosterone acetate. Corticosterone was extracted from plasma following manufacturer's protocol, by mixing 10μL of plasma with 10μL of diluted steroid displacement reagent (SDR; 1:100 dilution with deionised water) in sterile glass tubes for 5min at room temperature. Following this, 480μL assay buffer 15 (AB15) was added to give a final dilution of 1:50 for plasma sample. Samples were transferred to a fume hood where 3mL ethyl acetate (Sigma) was added to glass tubes and vortexed thoroughly. The clear upper organic layer was transferred to a fresh glass tube. The ethyl acetate was evaporated under nitrogen at 60°C. Samples and standards were prepared and assayed according to the manufacturer's instructions. The plate was read on a microplate reader (Magellan, TECAN) with optical density measurements at 405 nm, and corrected with 550 nm. The extraction efficiency was

assessed for each extraction by calculating the corticosterone concentration of a known sample following extraction. The average extraction efficiency throughout the experiments carried out was 82 ± 7%.

Copeptin & aldosterone measurement. Mice were euthanised by decapitation (between 0715 and 0800 local time), trunk blood was collected on ice, and separated by centrifugation and stored at -80ºC. After a single thaw of all samples, copeptin (CEA365Mu; Cloud-Clone Corp., USA; lower limit of detection 9pg/ml) and aldosterone (ADI-900-173; Enzo Life Sciences, UK; sensitivity of detection 4.7pg/ml) and were measured by commercial ELISA. Antibody-based methods of aldosterone can over-estimate aldosterone due to cross-reactivity with corticosterone and other steroids. The assay used has the following cross reactivity with other steroids: 11-Deoxycorticosterone (0.3%), Corticosterone (0.19%), Progesterone (0.20%), and <0.001% for Cortisol, DHT, Estradiol, Testosterone. The expression of renin in kidney homogenate was also measured to confirm suppression of the renin-angiotensin-aldosterone system with high salt intake.

Corticosterone Binding Globulin binding capacity. Terminal plasma samples were diluted (1:100) and stripped of endogenous steroids using dextran-coated charcoal. To calculate CBG specific binding, non-specific binding had to be subtracted from total binding. To measure total binding, samples were incubated with radioactively labelled corticosterone ([1,2,6,7-3H]corticosterone). To measure non-specific binding, samples were first incubated with high concentrations of unlabelled corticosterone in addition to radioactively labelled corticosterone. This meant that it was likely that the [1,2,6,7-3H]corticosterone would bind to unsaturated non-specific binding proteins. Free [1,2,6,7-3H]corticosterone was removed by further incubation with dextran-coated charcoal. The remaining radioactively labelled corticosterone bound to CBG was quantified by scintillation spectrophotometry. The maximal binding capacity (Bmax), measured in disintegrations per minute, was estimated using non-linear regression.

RNA isolation and quantitative PCR. Tissues were snap frozen in dry ice and stored at -80ºC.

RNA was extracted from mouse tissue, using the RNeasy Mini Kit (Qiagen, Hilden, Germany), by adding 300 – 600 µL RLT buffer (from the RNeasy Mini Kit) and a 5 mm stainless steel bead (Qiagen) to a 2 mL tube containing specific mouse tissue for homogenization, carried out using TissueLyser II (Qiagen) at 30 Hz for 1 min. The volume of RLT buffer was dependent on tissue weight as per manufacturer's instructions. Samples were centrifuged for 3 min at 8000 x g, and supernatant mixed with 1 volume of 70% ethanol (50% ethanol for liver tissue) and transferred to a spin column. Various spin steps and a DNAase step (RNase-free DNase Set, Qiagen) were then carried out according to manufacturer's protocol before RNA was eluted in 30-50 µL RNase free water (Thermo Fisher).

RNA concentrations, in ng/mL, were measured using a nanodrop 1000 spectrophotometer (Thermo Fisher). RNA integrity was assessed using automated electrophoresis (Bioanalyzer 2100, Agilent, CA, USA). The software assigns an RNA integrity number (RIN) to a total RNA sample, which is an objective metric of RNA quality. RIN ranges from 1 (completely degraded RNA) to 10 (highly intact RNA). RNA samples of a RIN of 7 or above were taken forward for reverse transcription.

cDNA was synthesised from 500ng of RNA, using the High Capacity cDNA Reverse Transcription (RT) kit (Applied Biosystems, CA, USA), where 10µL of 2x RT buffer and 1 µL of 20x enzyme mix were added to the diluted RNA and made up to 20 µL with nuclease free water. This was then reverse transcribed on a Veriti Thermal Cycler (Applied Biosystems). Negative controls were included, where the reverse transcriptase enzyme or RNA was replaced with an equal volume of water.

Primers for RT quantitative real-time polymerase chain reaction (RT-qPCR) were designed using the Universal Probe Library (UPL) Assay Design Centre. All designed UPL primers were synthesised by Integrated DNA technologies (IDT, IA, USA). Primers were designed where the amplicon spanned an intron, excluding the first or last intron. The resulting cDNA from the RT reaction was amplified and quantified using qPCR. Equal volumes of cDNA from samples were pooled together to construct an 8 point standard curve. The top standard of pooled samples were diluted 1:5 with nuclease-free water before serial dilutions (1:2) were carried out for standards 2-7. The final standard was used as a blank (nuclease-free water). The remaining cDNA was diluted 1:40 to fit in the middle of the standard curve. Sample/standard (2µL) was added to 8 µL of the qPCR master mix in triplicate in a 384 well plate. Primers and dual hybridisation probes used in RT-qPCR reactions are illustrated in **Supplemental table 1**. Roche discontinued the UPL in January 2020 and additional qPCR experiments performed during manuscript revision used Qiagen QUantiTect Primer Assays for *Tonebp* (Mm_Nfat5_1_SG Cat no. QT01053647), Pc (Pyruvate carboxylase Mm_Pcx_1_SG Cat no. QT00107457) and *Pepck* (Phosphoenolpyruvate Carboxykinase 1 Mm_Pck1_1_DG Cat no. QT00153013)

For all qPCR assays, the Roche LightCycler 480 (Roche Diagnostics Ltd., Basel, Switzerland) was used to perform thermocycling and fluorescence detection, using the specific cycling conditions. Threshold cycle/crossing point (Cq) values for each well were quantified, using the automated LightCycler 480 software. Each triplicate was analysed and excluded if the Cq SD was > 0.5. For each gene of interest and reference gene, the standard curve was analysed and had to meet criteria of standard curve efficiency being within the range of 1.7- 2.1 and error < 0.05. Gene expression was analysed as relative expression as each gene was normalised against the mean expression of a minimum of 2 reference genes. A panel of reference genes were tested for each tissue (including *Actb*, *Gapdh*, *Hprt, Rn18s* and *Tbp*) and appropriate reference genes were selected for each tissue on the basis that they did not differ between treatment groups. The reference genes were: *Actb* and *Tbp* for adrenal, hippocampus and anterior pituitary; *Actb* and *Hprt* for liver; *Actb* and *Rn18s* for kidney cortex/medulla and aorta; *Actb* and *Gapdh* for heart; *Gapdh* and *Tbp* for hypothalamus. Data were then log transformed to give symmetry and normality tests were conducted. RT-qPCR data were handled in accordance to the MIQE guidelines. Q values adjusted p values controlling for the false discovery rate (FDR), were also reported to adjust for multiple comparisons in gene expression data for tissues.

Table S1: List of Primers

**Gene**	Protein	Forward primer	Reverse Primer	UPL probe no.
Actb	βactin	ctaaggccaaccgtgaaaag	accagaggcatacagggaca	64
Gapdh	GAPDH	gggttcctataaatacggactgc	ccattttgtctacgggacga	52
Hprt	HPRT	tcctcctcagaccgctttt	aacctggttcatcatcgctaa	95
Rn18S	18S rRNA	gccgctagaggtgaaattctt	cgtcttcgaacctccgact	93
Tbp	TBP	gggagaatcatggaccagaa	gatgggaattccaggagtca	97
Agt	AGT	cggaggcaaatctgaacaac	tcctcctctcctgctttgag	84
Avp	AVP	gctgccaggaggagaactac	aaaaccgtcgtggcactc	88
Avpr1a	V1aR	gggataccaatttcgtttgg	aagccagtaacgccgtgat	31
Avpr1b	V1bR	cattgtgctggcctacattg	tggtgaaagccacattggta	104
Avpr2	V2R	cctggtgtctaccacgtctg	gcaccagactggcatgtatct	27
Crh	CRH	gaggcatcctgagagaagtcc	atgttaggggcgctctcttc	34
Crhr1	CRHR1	gggccattgggaaacttta	atcagcaggaccaggatca	81
Cyp11b1	CYP11B1	gccatccaggctaactcaat	cattaccaaggggggttgatg	11
Cyp11b2	CYP11B2	aagctcagacttggtgcttca	cggcccatggagtagagata	3
Fkbp5	FKBP5	aaacgaaggagcaacggtaa	tcaaatgtccttccaccaca	97
Hsd11b1	11βHSD1	tctacaaatgaagagttcagaccag	gccccagtgacaatcacttt	1
Hsd11b2	11βHSD2	cactcgaggggacgtattgt	gcaggggtatggcatgtct	26
Mc2r	MC2R	caccacaatcctctaccctca	cctctccttggctttgtcac	96
Nr3c1	GR	gacgtgtggaagctgtaaagt	catttcttccagcacaaaggt	56
Nr3c2	MR	ttcggagaaagaactgtcctg	cccagcttctttgactttcg	50
Pomc	POMC	agtgccaggacctcacca	cagcgagaggtcgagtttg	62
Ren	renin	gggaggcagggcctacac	ctctcctgttgggatactgtagca	*
Serpina6	CBG	ccaccaaagacactcccttg	ggtgtacaggagggccatt	40
Srd5a1	5α-reductase1	gggaaactggatacaaaataccc	ccacgagctccccaaaata	41
StAR	StAR	aaggctggaagaaggaaagc	ccacatctggcaccatctta	2

Abbreviations: GAPDH: glyceraldehyde 3-phosphate dehydrogenase, HPRT: hypoxanthine-guanine phosphor-ribosyltransferase, rRNA: ribosomal RNA, TBP: TATA-binding protein, AGT: angiotensinogen, AVP: vasopressin, V1aR: vasopressin receptor 1A, V1bR: vasopressin receptor 1B, V2R: vasopressin receptor 2, CRH: corticosterone releasing hormone, CRHR1: CRH receptor 1, CYP11B1/2: cytochrome P450, family 11 subfamily b polypeptide 1/2, FKBP5: FK506 binding protein 51, 11βHSD1 or 2: *11β*-Hydroxysteroid dehydrogenase type 1 or 2, MC2R: melanocortin receptor 2, GR: glucocorticoid receptor, MR: mineralocorticoid receptor, POMC: pro-opiomelanocortin, CBG: corticosteroid binding globulin, StAR: steroidogenic acute regulatory protein.
* performed using PowerUp SYBR Green (Applied Biosystems, UK).

Figure S1. Male C57BL6/J mice were fed either a 0.3% Na diet (Control, open circles) or a 3% Na diet (High Salt; grey circles) for 14 days. A) Adrenal gland weight (n=8/8) and (B) adrenal gland mRNA abundance for *Cyp11b1* (n=8/8) and *Mcr2* (n=8/8). Individual values are shown with group mean±SD; statistical comparisons were made using Student's unpaired *t* test with two-tailed p values stated.

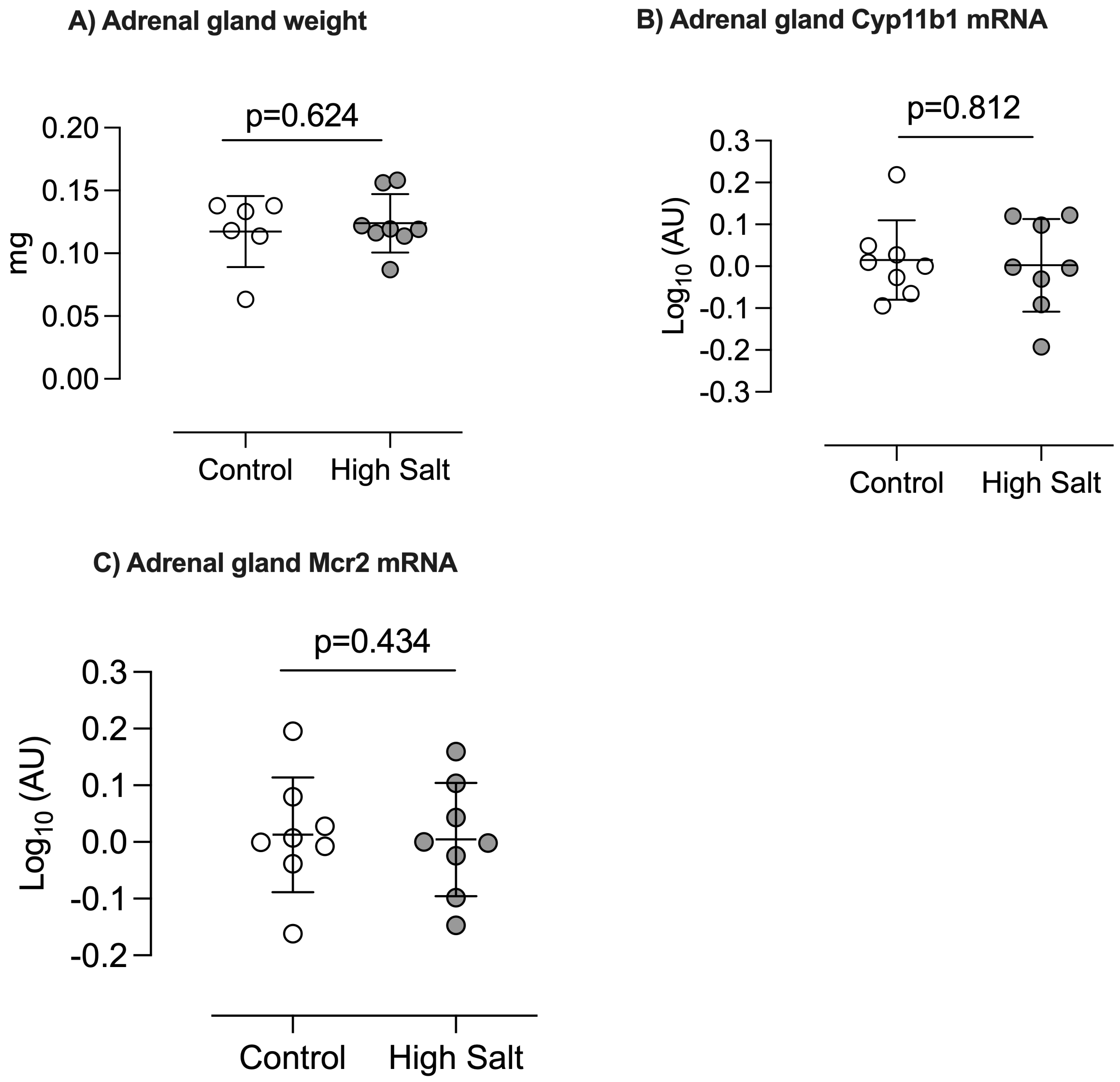

Figure S2. Male C57BL6/J mice were fed either a 0.3% Na diet (Control, open circles) or a 3% Na diet (High Salt; grey circles) for 14 days and the following measured: A) hepatic angiotensinogen (*Agt) mRNA;* B) renin (*Ren*) mRNA in kidney cortex; C) adrenal gland aldosterone synthase mRNA (*Cyp11b2);* D) plasma aldosterone and E) mRNA abundance for the mineralocorticoid receptor (*Nr3c2)* in kidney cortex and F) kidney medulla. Individual values are shown with group mean±SD; statistical comparisons were made using Student's unpaired *t* test with two-tailed p values stated.

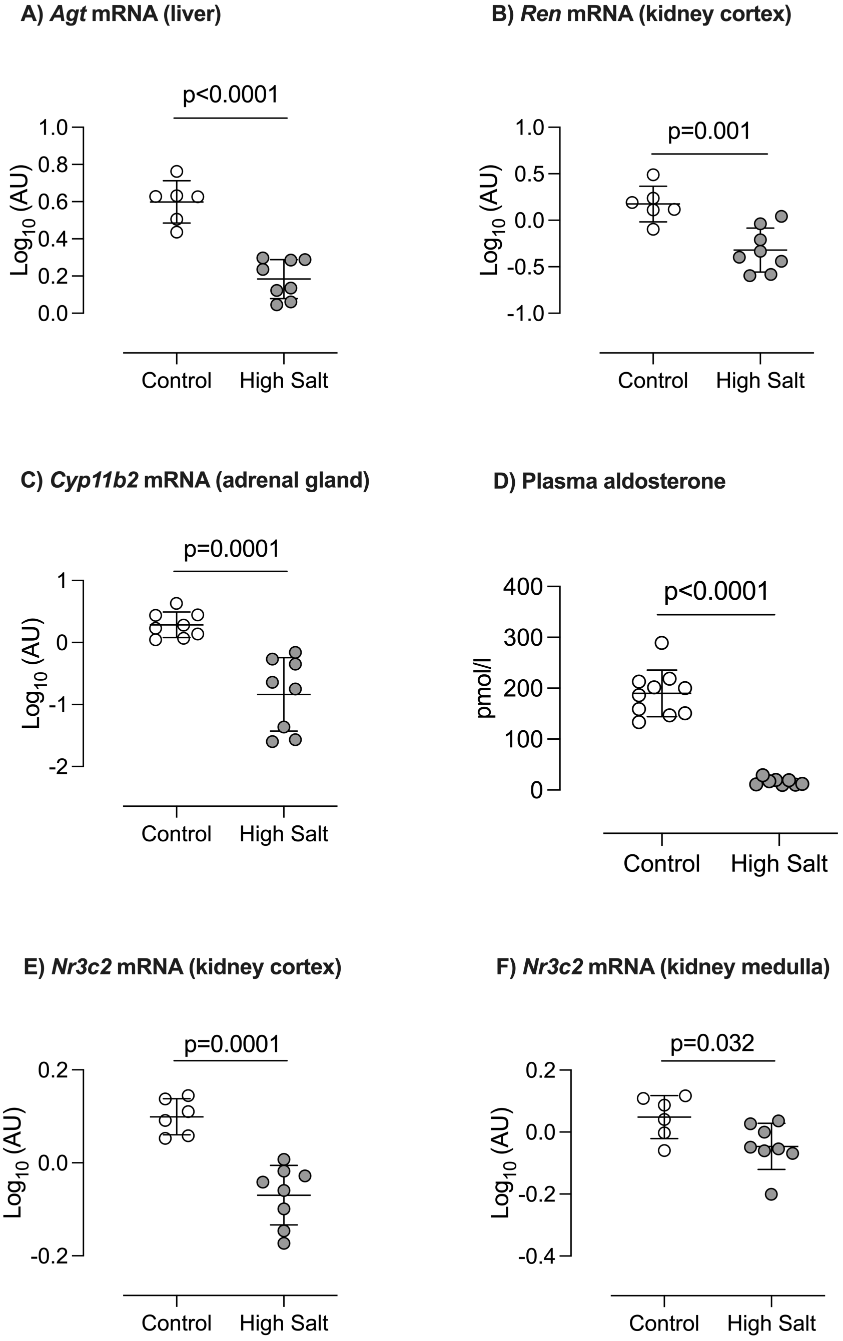

Figure S3. A) Plasma CBG binding capacity (n=7/7); B) liver mRNA abundance for *SerpinA6* (n=6/8) from male C57BL6 mice fed either 0.3% Na diet (Control, open circles) or 3% Na diet (High Salt; grey circles) for 7 days. Individual values are shown with group mean±SD; statistical comparisons were made using Student's unpaired *t* test with two-tailed p values stated.

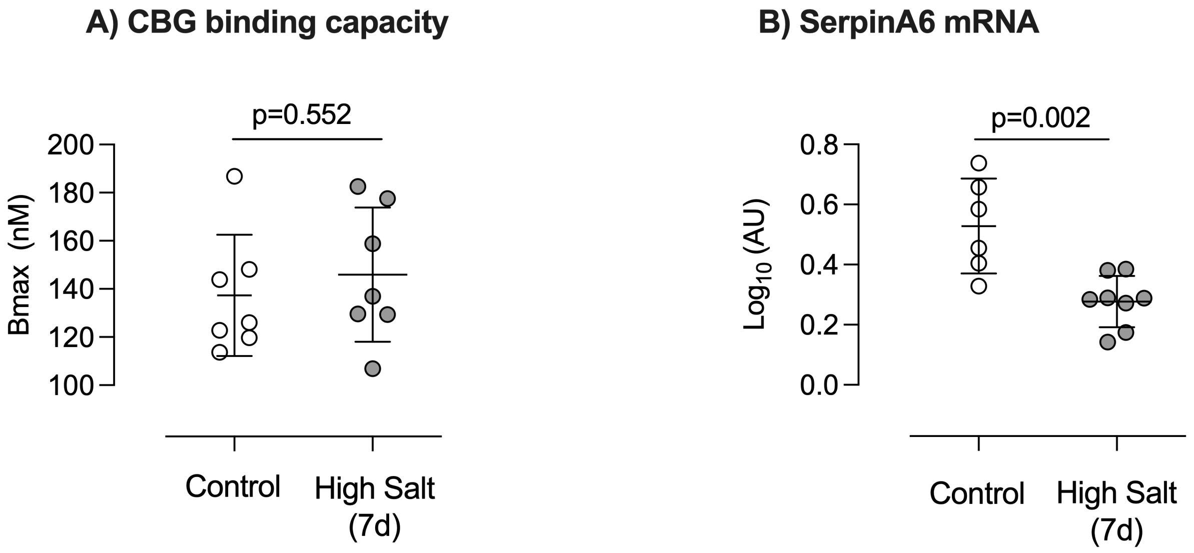

Figure S4. mRNA abundance in of mRNA for 11β hydroxysteroid dehydrogenase (*Hsd11b1*) in hippocampus from mice fed either 0.3% Na diet (Control, open circles) or 3% Na diet (High Salt; grey circles) for 14 days. Individual values are shown with group mean±SD; statistical comparisons were made using Student's unpaired *t* test with two-tailed p values stated.

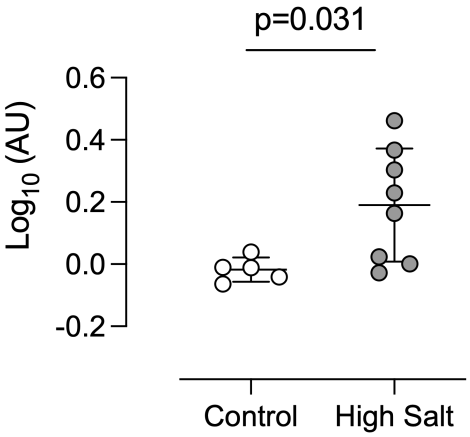

Figure S5. mRNA abundance in liver of A) FK506 binding protein 5 (*Fkbp5)*; B) glucocorticoid receptor (*Nr3c1*) and C) 11β hydroxysteroid dehydrogenase type 1 (*Hsd11b1)* from mice fed either 0.3% Na diet (Control, open circles) or 3% Na diet (High Salt; grey circles) for 14 days. Individual values are shown with group mean±SD; statistical comparisons were made using Student's unpaired *t* test with two-tailed p values stated.

A) Liver *Fkbp5* mRNA

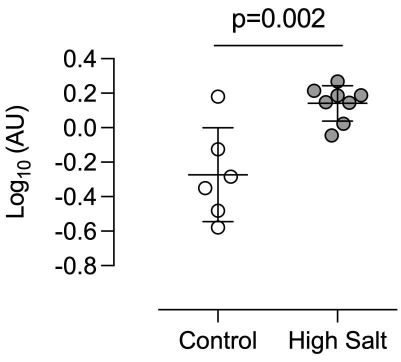

B) Liver *Nr3c1* mRNA

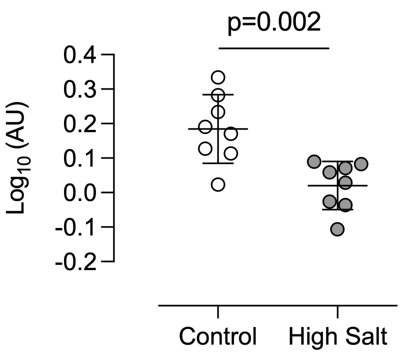

C) Liver Hsd11b1 mRNA

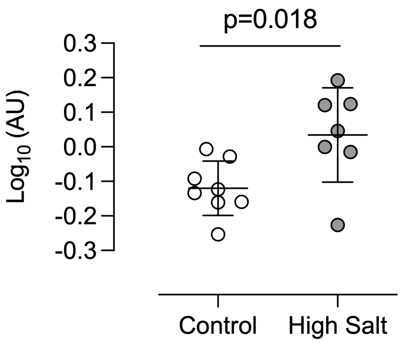

Figure S6. mRNA abundance in kidney cortex and medulla of A) FK506 binding protein 5 (*Fkbp5)*; B) glucocorticoid receptor (*Nr3c1*); C) 11β hydroxysteroid dehydrogenase type 1 (*Hsd11b1)* and D) 11β hydroxysteroid dehydrogenase type 2 (*Hsd11b2)* from mice fed either 0.3% Na diet (Control, open circles) or 3% Na diet (High Salt; grey circles) for 14 days. Individual values are shown with group mean±SD; statistical comparisons were made using Student's unpaired *t* test with two-tailed p values stated.

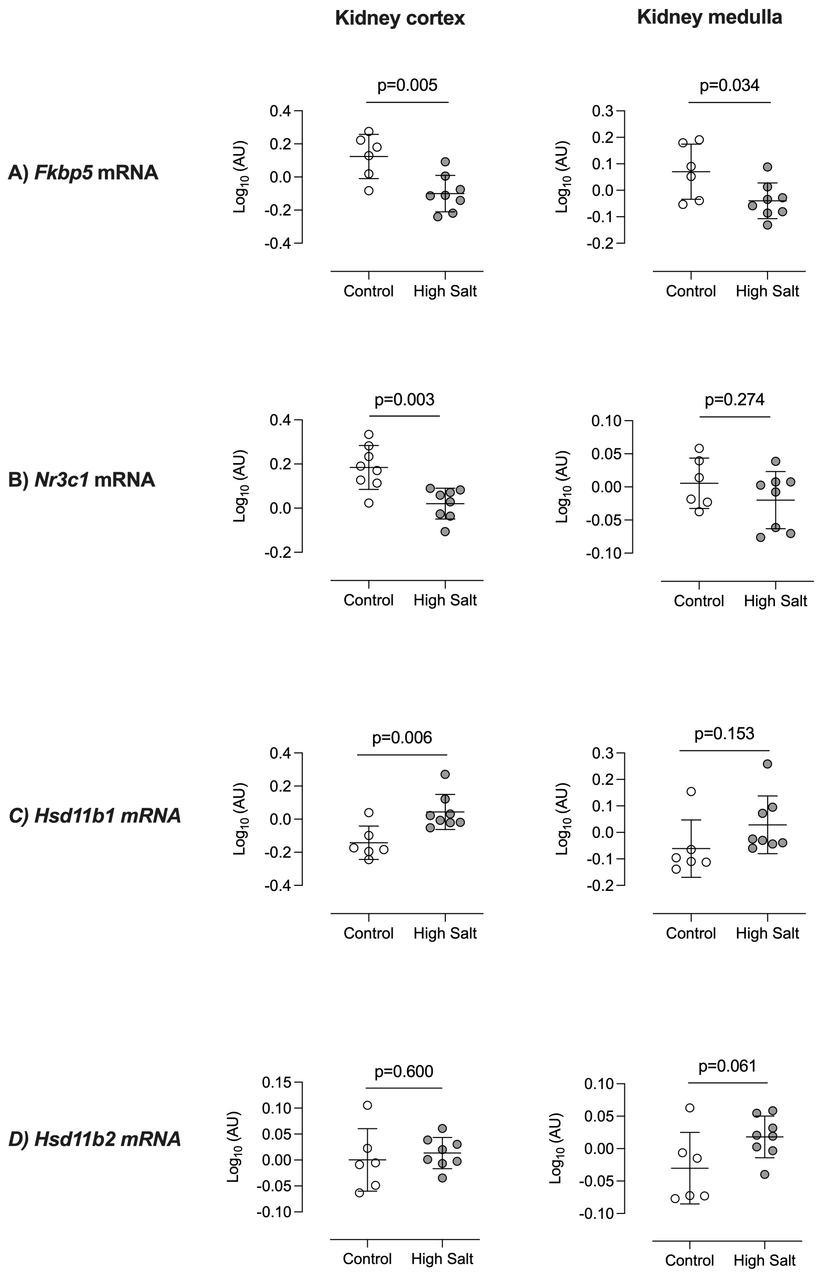

Figure S7. mRNA abundance in heart and aorta of A) FK506 binding protein 5 (*Fkbp5)*; B) glucocorticoid receptor (*Nr3c1*) and C) 11β hydroxysteroid dehydrogenase type 1 (*Hsd11b1)* from mice fed either 0.3% Na diet (Control, open circles) or 3% Na diet (High Salt; grey circles) for 14 days. Individual values are shown with group mean±SD; statistical comparisons were made using Student's unpaired *t* test with two-tailed p values stated.

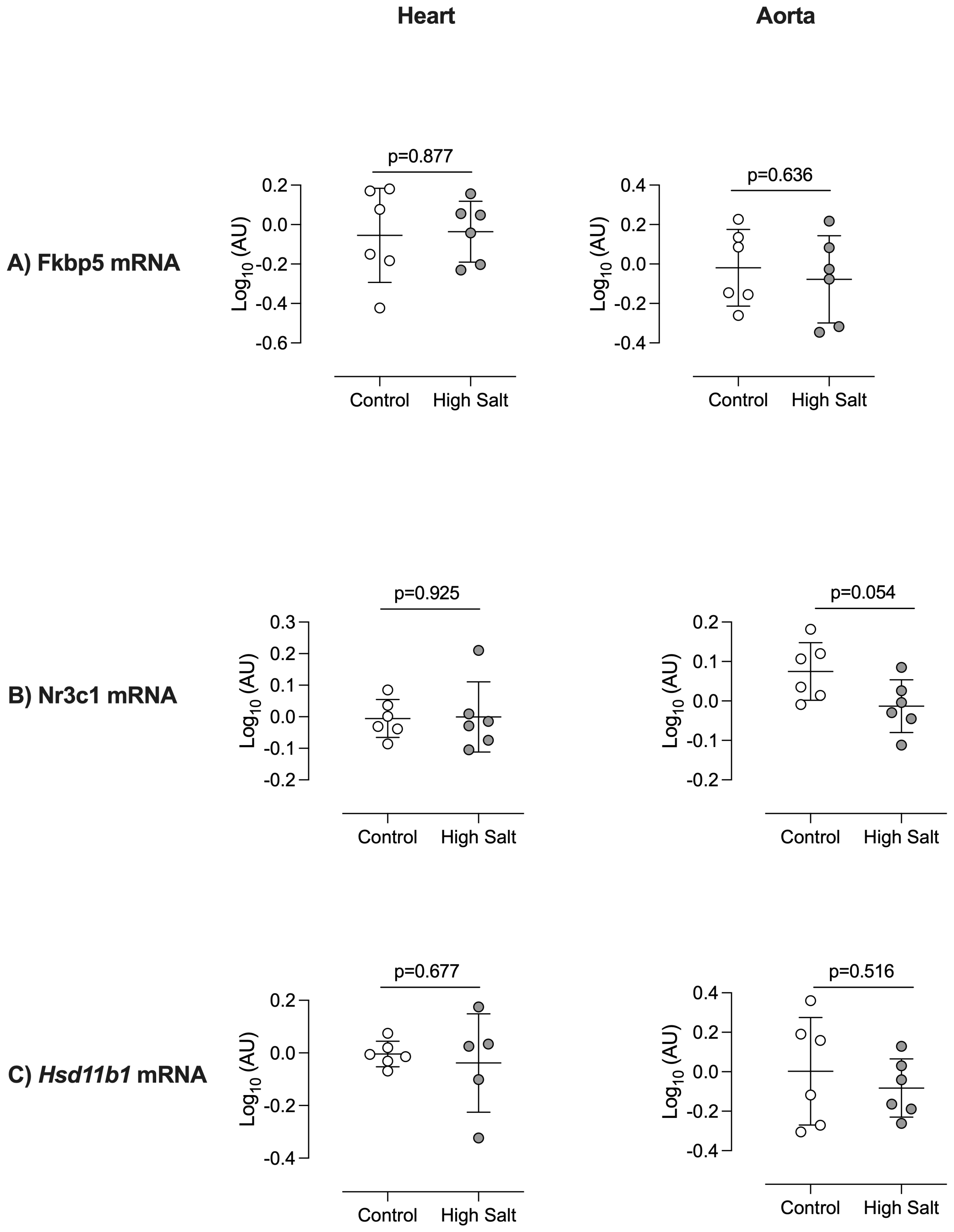

Figure S8. mRNA abundance in adrenal gland of A) FK506 binding protein 5 (*Fkbp5)*; B) glucocorticoid receptor (*Nr3c1*) and C) 11β hydroxysteroid dehydrogenase type 1 (*Hsd11b1)* from mice fed either 0.3% Na diet (Control, open circles) or 3% Na diet (High Salt; grey circles) for 14 days. Individual values are shown with group mean±SD; statistical comparisons were made using Student's unpaired *t* test with two-tailed p values stated.

Adrenal gland

A) *Fkbp5* mRNA

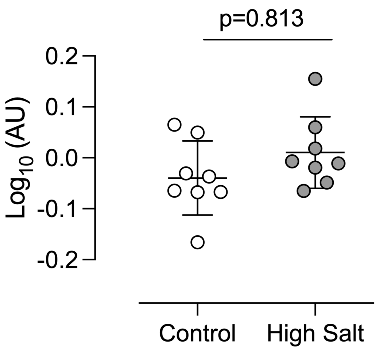

B) *Nr3c1* mRNA

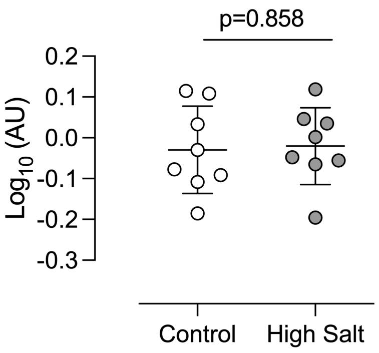

C) *Hsd11b1* mRNA

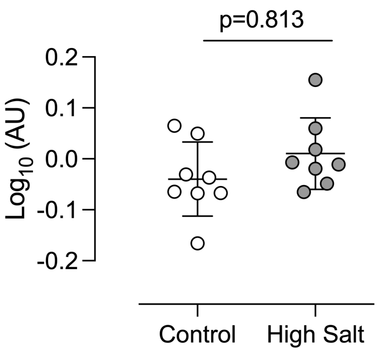

Figure S9. Plasma concentration of A) sodium and B) urea and mRNA abundance in liver and kidney cortex of C) Phosphoenolpyruvate Carboxykinase 1 (*Pck1)*; and D) pyruvate carboxylase (*Pc*) from mice fed either 0.3% Na diet (Control, open circles) or 3% Na diet (High Salt; grey circles) for 14 days. Individual values are shown with group mean±SD; statistical comparisons were made using Student's unpaired *t* test with two-tailed p values stated.

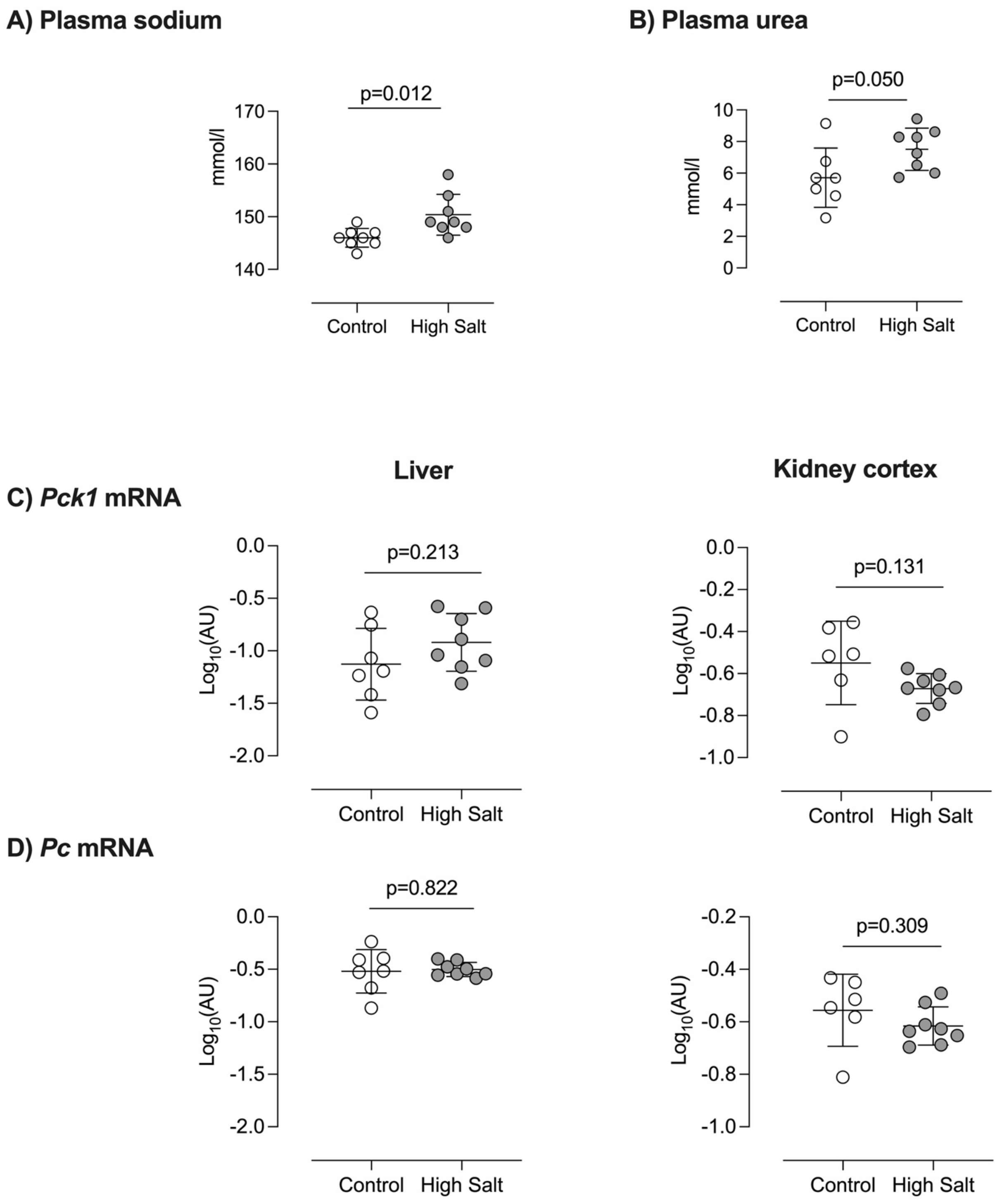

Supplement: cvac160_Supplementary_Data [file cvac160_supplementary_data.pdf]
